# Efficient structure-factor modeling for crystals with multiple components

**DOI:** 10.1107/S205327332300356X

**Published:** 2023-06-20

**Authors:** Pavel V. Afonine, Paul D. Adams, Alexandre G. Urzhumtsev

**Affiliations:** a Molecular Biophysics and Integrated Bioimaging Department, Lawrence Berkeley National Laboratory, One Cyclotron Road, Berkeley, California 94720, USA; b Department of Bioengineering, University of California Berkeley, Berkeley, California, USA; c Centre for Integrative Biology, Institut de Génétique et de Biologie Moléculaire et Cellulaire, CNRS-INSERM-UdS, 1 rue Laurent Fries, BP 10142, Illkirch, 67404, France; d Faculté des Sciences et Technologies, Université de Lorraine, BP 239, Vandoeuvre-les-Nancy, 54506, France; University of Warsaw, Poland

**Keywords:** structure factors, multiple components, scattering functions, bulk solvent, refinement, density maps

## Abstract

Diffraction intensities from a crystallographic experiment include contributions from the entire unit cell of the crystal: the macromolecule, the solvent around it and eventually other compounds. These contributions cannot typically be well described by an atomic model alone, *i.e*. using point scatterers. Indeed, entities such as disordered (bulk) solvent, semi-ordered solvent (*e.g.* lipid belts in membrane proteins, ligands, ion channels) and disordered polymer loops require other types of modeling than a collection of individual atoms. This results in the model structure factors containing multiple contributions. Most macromolecular applications assume two-component structure factors: one component arising from the atomic model and the second one describing the bulk solvent. A more accurate and detailed modeling of the disordered regions of the crystal will naturally require more than two components in the structure factors, which presents algorithmic and computational challenges. Here an efficient solution of this problem is proposed. All algorithms described in this work have been implemented in the computational crystallography toolbox (*CCTBX*) and are also available within *Phenix* software. These algorithms are rather general and do not use any assumptions about molecule type or size nor about those of its components.

## Abstract 2

A multi-component description of the unit-cell content is introduced. Efficient algorithms to define the contribution of these components to structure factors are described and implemented in *CCTBX* and *Phenix*.

## 1. Introduction

Experimentally measured intensities of the crystallographic structure factors reflect the content of the whole crystal. Therefore, accurate modeling of the crystal content requires corresponding structure factors to account for all scattering matter present in the unit cell. This includes bulk solvent and other semi-ordered or disordered entities, such as disordered loops or ligands. Currently, crystallographic packages such as *SHELXL* (Sheldrick, 2008 ▸), *CNS* (Brünger *et al.*, 1998 ▸), *REFMAC* (Murshudov *et al.*, 2011 ▸), *Phenix* (Liebschner *et al.*, 2019 ▸) employ the two-component model for the total structure factor:

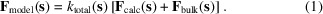

Here 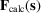 is the contribution from all ordered atoms (macromolecule, solvent, ligands) and **s** represents a reciprocal-space vector. 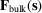 accounts for the bulk solvent contribution using one of the available models: exponential (Moews & Kretsinger, 1975 ▸; Tronrud, 1997 ▸), radial-shell (Jiang & Brünger, 1994 ▸), flat with exponential (Jiang & Brünger, 1994 ▸) or per-resolution scalar scale (Afonine *et al.*, 2013 ▸). 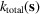 is the overall anisotropic resolution-dependent scale factor. A similar approach, referred to as *PLATON* *SQUEEZE* (Spek, 2015 ▸), is used in small-molecule crystallography, where the contribution of the disordered content of the unit cell is explicitly calculated and added to the total model structure factors. *BUSTER* (Roversi *et al.*, 2000 ▸; Blanc *et al.*, 2004 ▸) uses the three-component model

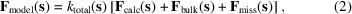

where 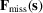 describes components other than bulk solvent that cannot be modeled with individual atoms (such as the disordered part of a macromolecule or ligands).

Below we propose a more general definition of the total model structure factor:

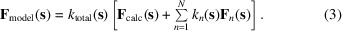

Here 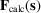 are calculated on the absolute scale from the principal part of the model, *e.g*. atomic model. Terms 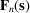 stand for structure factors arising from other (for example, non-atomic) components added to the sum with some scale factors 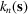 . In the simplest case where there is no prior knowledge available about these non-atomic components, 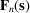 can be the structure factors calculated from a binary 0–1 mask of the component *n*, with 1 inside the region and 0 outside, similar to the flat bulk solvent model (Jiang & Brünger, 1994 ▸). However, any other model considering the contribution from different parts of the crystal as independent is applicable. When some prior information is available, then more sophisticated 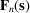 models can be used (Blanc *et al.*, 2004 ▸). The number *N* is not specific for the algorithms and is defined by a particular problem. Practically, we expect it to vary from a few up to several hundreds.

The values of the resolution-dependent scale factors 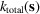 and 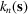 can be obtained by fitting 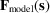 to the observed structure-factor amplitudes 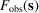 . At this stage, we consider all structure factors as constants and search only for the scale factors.

When 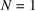 , *i.e.* when a single bulk solvent contribution is considered, a possible solution has been reported in detail and implemented in *CCTBX* and *Phenix* (Afonine *et al.*, 2013 ▸). When 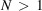 , a fast, robust and memory-efficient algorithm is needed. Here we propose four possible algorithms, discuss the strengths and weaknesses of each of them, and argue for one to be used as a default choice.

## 2. Methods

### 2.1. Common considerations

Assuming 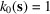 and denoting 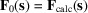 , expression (3) can be rewritten as

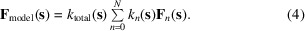

Here 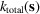 is the unknown overall anisotropic scale factor (Sheriff & Hendrickson, 1987 ▸; Afonine *et al.*, 2013 ▸), 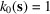 and 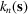 for 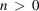 are unknown scale functions. We suppose that 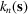 are smooth isotropic functions of the resolution, *i.e.* 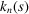 where 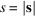 . No particular analytical shape is assumed for 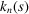 , as argued by Urzhumtsev & Podjarny (1995 ▸) and Afonine *et al.* (2013 ▸).

The functions 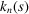 vary slowly within sufficiently thin resolution shells. The resolution shells are defined uniformly in the logarithmic resolution scale (Urzhumtsev *et al.*, 2009 ▸; Table 1 in Afonine *et al.*, 2013 ▸) with two additional and somewhat contradictory requirements: the shells should be thin enough to consider scale factors 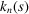 as constant inside each shell and they should contain a sufficient number of reflections to make determination of 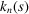 values statistically valid. The latter condition concerns mostly the lowest-resolution shells.

If all the *N* components have the same scattering function (form factor), then (4) can be simplified,

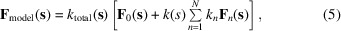

where scale factors 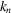 are independent of resolution and can be thought of as occupancy factors of respective components, and 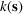 is an overall resolution-dependent scale factor for all the components. An advantage of (5) with respect to (4) is that it uses a single parameter 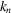 for all structure factors 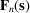 , and the total number of independent parameters reduces from 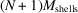 to 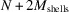 , where 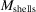 is the number of resolution shells.

### 2.2. Initialization

The scaling procedure is iterative and initiated with the observed structure-factor amplitudes or intensities, 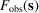 or 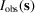 , and a set of 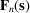 . The initial values of 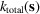 and of 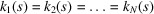 are obtained as described by Afonine *et al.* (2013 ▸) considering contributions from all non-atomic components as a single one. Once all components 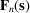 are accounted for, the overall scale factor 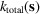 can be updated.

Observed amplitudes 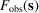 or intensities 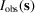 and scaled 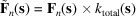 are the inputs to each of four algorithms, referred to below as algorithms 1–4. Then, calculations of improved 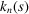 values are performed independently in resolution shells. The procedure is repeated iteratively, until convergence, with 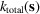 and 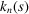 being updated at each iteration.

In what follows, to simplify expressions, we omit the index of the resolution shell when this does not lead to confusion.

### 2.3. Algorithms to search for the scale coefficients

#### 2.3.1. Algorithm 1: sequential search

In algorithm 1 each component 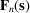 is added to 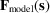 sequentially one at a time followed by the update of 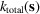 . For each new 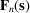 that is being added the scale factors 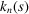 are computed as described by Afonine *et al.* (2013 ▸). This means that at each iteration the procedure of Afonine *et al.* (2013 ▸) is applied *N* times, equal to the number of components, which makes the procedure very expensive computationally. Also, errors in initially roughly estimated parameters such as 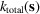 can propagate into 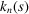 of components being added and that can result in the failure of the whole procedure.

#### 2.3.2. Algorithm 2: iterative one-step search

Considering all coefficients 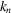 in each resolution shell as constants, this algorithm searches simultaneously for their values, minimizing the residual

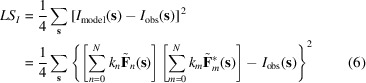

with respect to 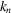 . Here the outer sums are calculated over reflections of the given shell. Developing the expression in curly brackets and swapping the sums over components and over reflections, this expression can be rewritten as

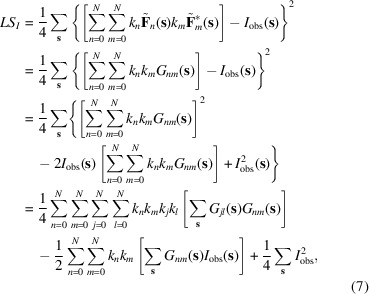

where

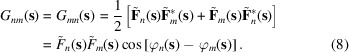




The model values to be compared with the observed intensities (6) include not only the intensities from the individual components, 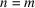 , but also the cross-terms mixing unscaled structure factors from two components, 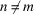 . Being a half-sum of two complex conjugates (8), coefficients 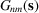 describing these cross-terms are real numbers.

The polynomial of the fourth degree (7) with respect to individual scale factors 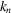 can be minimized using a standard approach, *e.g.* L-BFGS (Liu & Nocedal, 1989 ▸). Similar to other gradient-based algorithms for a local minimization, it is an iterative procedure which requires the initial values for refinable variables to be reasonably close to the expected solution, as well as all partial derivatives with respect to these variables. Depending on the number of refinable variables and the proximity of their initial values to the expected solution, several (typically between ten and 100) iterations of minimization are typically required. The derivatives of 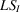 with respect to 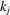 , 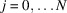 , required by the minimizer, are

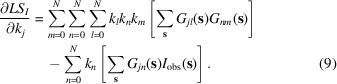




#### 2.3.3. Algorithm 3: non-iterative two-step search

In this algorithm, instead of using iterative minimization methods, we search for the minimum of (6) analytically, which does not require an estimate of initial values for 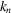 . First, we introduce (*N* + 1)^2^ intermediate parameters,

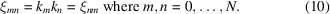

We start from the search for their values that we decompose later into individual coefficients 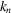 .

Rewriting the function (6) using new variables (10) makes it a quadratic function of these new variables,

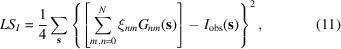

which we minimize with respect to 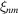 . The minimum of 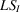 can be found as a solution of a system of linear equations with respect to these unknowns. After excluding the redundant variables due to the commutativity property, 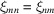 , we stay with 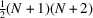 equations 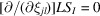 for the independent variables 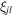 , 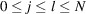 :

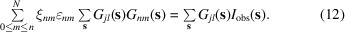

Here 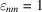 if 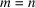 and 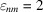 otherwise, as this comes after swapping the order of summation in derivatives of (11) and putting together the terms with the indices *mn* and *nm*. This is a system of linear equations that can be solved using a standard approach (for example, Meckes & Meckes, 2018 ▸).

Solution of (12) yields 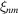 values (10), 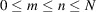 , which now allows one to search for 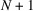 scale coefficients 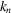 by minimizing the following residual:

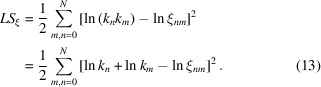

Using logarithms rather than the values themselves in (13) allows us to find the minimum of (13) with respect to 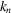 analytically as a solution of the system of linear equations

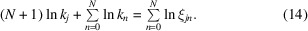

This gives

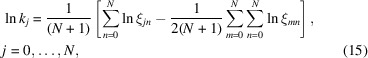

where recovering 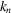 from 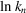 is trivial.

While this algorithm requires neither iterations nor initial values of the scale factors, its serious disadvantage is the large dimension of the system of equations (12), the need to use a square matrix of the dimension 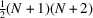 , and sensitivity to rounding errors. This makes it impractical when applied to real structures and we describe it here for the sake of completeness.

#### 2.3.4. Algorithm 4: iterative phased search

With this algorithm, we try to avoid both an iterative minimization of a function of many variables (algorithm 2) and the use of a large system of equations (algorithm 3). To do so, instead of comparison of intensities, we compare structure factors as complex values. The generally unknown phase values 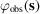 can be approximated as those of the model structure factors (4),

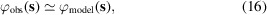

which is a reasonable assumption for a nearly finalized model, the scenario when the multi-component model is expected to be used. We express the best fit of the complex structure factors as a function to be minimized with respect to 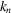 ,

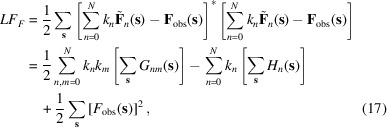

where 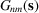 are defined previously by (8) and 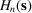 are defined similarly as

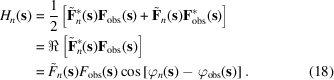




Minimization of (17) results in a system of *N* + 1 linear equations with respect to 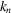 :

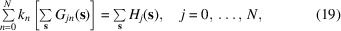

which, similarly to (12), can be solved using a standard approach. Several iterations, typically up to a few dozens, may be required to solve (17), with each iteration improving model structure factors (4) and respective phase values (16) and updating 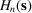 (18).

## 3. Testing algorithms 2 and 4

### 3.1. Generalities

As discussed in the Introduction, the multi-component model may be applied to the solution of various problems and, generally, it consists of two stages: (i) defining these components and calculation of structure factors from them, and (ii) combining these structure factors together into the total model structure factor (4). The first stage (defining components) is very problem specific. The components may arise as a result of annotation of macromolecular cavities (Matthews & Liu, 2009 ▸), or map analysis to find and model regions of semi-ordered lipid layers (Sonntag *et al.*, 2011 ▸), or from calculating blurred binary masks to account for the bulk solvent (Jiang & Brünger, 1994 ▸), or use a large-Gaussian model to approximate yet unmodelled parts of the macromolecule (Lunin *et al.*, 1995 ▸), and so on. The second stage (combining structure factors from multiple components) is not problem specific: it is independent of how the components and their structure factors were obtained. Since in this work we describe the algorithms that address the second stage, the test calculations described below have been done using a simple self-contained model to prove that the algorithms can find accurate values of the multi-component optimal scale functions 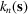 in (4). In what follows, we focus on algorithms 2 (iterative one-step search) and 4 (iterative phased search) as algorithms 1 (sequential search) and 3 (non-iterative two-step search) are much less likely to find practical application. Also, as stated in Section 2.2, during the search for the scale factors 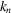 all structure factors, 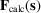 and 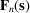 , remain unchanged.

### 3.2. Error-free test with a few components representing isolated regions inside a protein

To test the performance of these algorithms, the following numeric experiment was set up. The Ypd1p model [PDB (Protein Data Bank) code 1c03, Song *et al.*, 1999 ▸] was obtained from the PDB (Burley *et al.*, 2021 ▸) and the bulk solvent mask was calculated using the standard approach (Jiang & Brünger, 1994 ▸). This mask has one large isolated region that constitutes about 57% of the unit-cell volume (174 794 Å^3^) and six much smaller regions with the volume varying between 50 and 190 Å^3^. Each of these regions was considered as an individual solvent region with its own binary mask, 1 inside the region and 0 outside. The total model structure factor for this system was defined according to (3) as

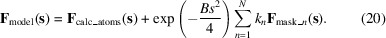

Here, 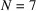 is the number of regions, and the exponential resolution-dependent scale factor was introduced similarly to the flat bulk solvent model to smooth the sharp boundaries of masks with the smearing *B* factor of 50 Å^2^ (Fokine & Urzhumtsev, 2002 ▸). Each region was assumed to have its own individual scale factor 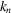 , and their values were assigned randomly in the range between 0 and 1. For each trial choice of 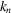 the corresponding set of structure factors (20) was calculated and their absolute values were then referred to as error-free ‘observable data’ 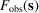 . These 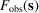 , 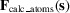 and the set of smeared 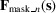 were subjected to algorithms 2 and 4 and the obtained values of 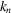 were compared with the known values using relative error as a measure. Additionally, the crystallographic *R* factor was calculated using the known exact 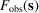 and model structure factors (2) calculated with 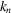 values recovered by one of the two algorithms. Since the outcome of the procedure can potentially depend on the choice of 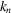 used to calculate 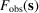 and the initial 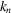 values used by algorithms, the procedure was repeated 1000 times, each time using the different set of 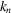 and varying the initial values for 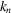 within about an order of magnitude from the known values. In all cases, both algorithms recovered the 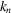 values almost exactly, within 0.0001% error, regardless of the choice of 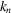 and the initial values.

### 3.3. Robustness with respect to errors in the atomic model

Additionally, the performance of the algorithms was assessed in the presence of random errors in atomic model coordinates using the same test setup as in Section 3.2.

Generally, the errors can be of several types (*e.g.* systematic, random) and have many sources, such as errors in atomic model parameters (coordinates, *B* factors, occupancies) or model incompleteness, as well as errors in experimental data (measurement errors, completeness). Here we only focus on removable model errors (Lunin *et al.*, 2002 ▸), which do not prevent the model eventually reproducing the experimental data accurately if all model parameters have their exact values. This is fundamentally different to the case of irremovable errors. An example of irremovable errors is crystal structure model incompleteness, when the model describes only a part of the entire unit-cell content. In this case no choice of model parameters can fully compensate for the missing scattering and the best fit of model parameters to the data does not necessarily lead to accurate model parameters, in fact, the opposite (Lunin *et al.*, 2002 ▸). This problem is typically addressed by the appropriate choice of refinement target function and not by the optimization procedure itself (Lunin *et al.*, 2002 ▸).

**Figure 1:**
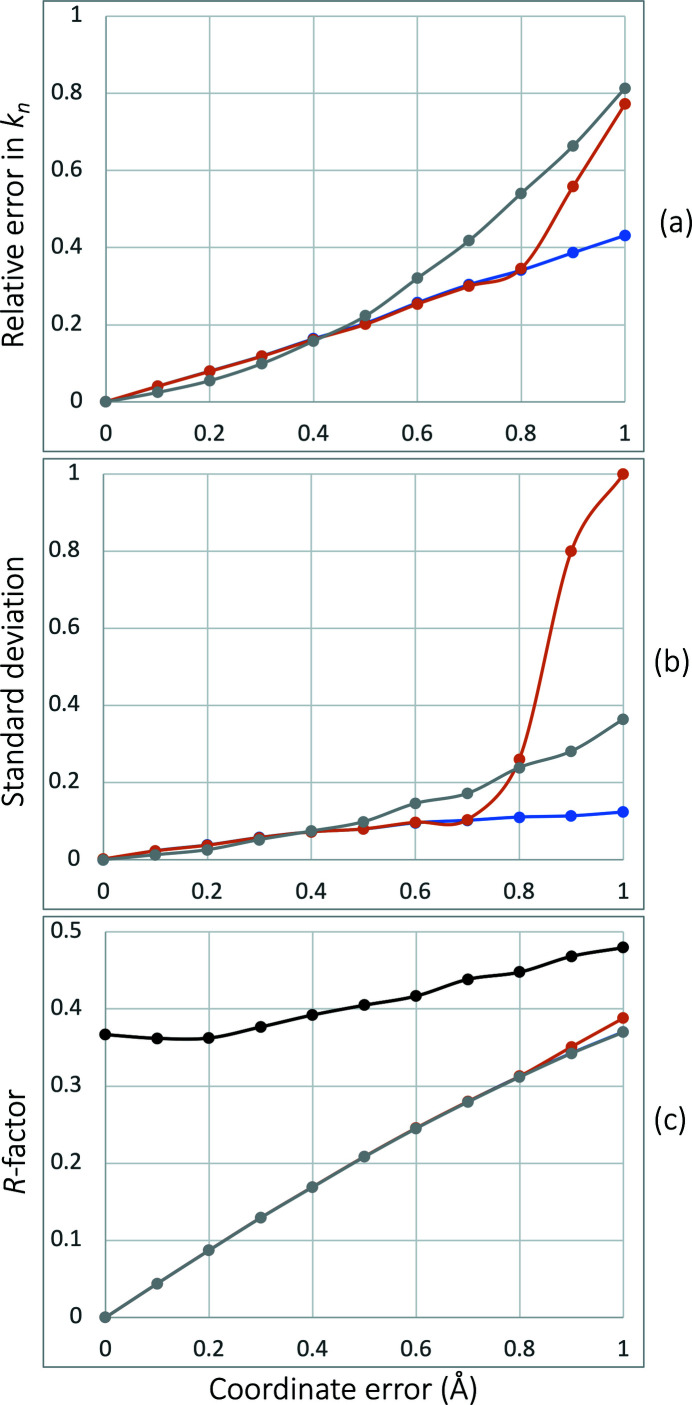
Relative mean error in 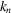 (*a*), its standard deviation (*b*) and (*c*) *R* factor between error-free simulated 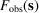 and 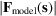 (20) computed from an atomic model with indicated mean coordinate errors using 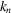 values recovered by algorithm 4 (gray), algorithm 2 without second derivatives (orange) and algorithm 2 using second derivatives (blue). The black line in (*c*) shows the initial *R* factor calculated assuming all 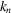 values are zero.

Provided the model completely describes the unit-cell content, errors in atomic coordinates are an example of removable errors that we consider in what follows. Also, simulation of random errors in atomic coordinates can be thought of as somewhat similar to the simulation of correlated random errors in the experimental data (Lunin *et al.*, 2002 ▸; Holton *et al.*, 2014 ▸). Thus, in the following test random errors of different magnitude were introduced to atomic coordinates leading to the root-mean-squared deviation (RMSD) between initial unperturbed and perturbed models in the range between 0 and 1 Å with a step of 0.1 Å. The unperturbed atomic model, the set of mask structure factors calculated for each of seven regions and the known values of 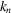 were used to generate 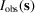 using formula (20). The perturbed model was used to calculate 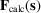 during the search. For each perturbation dose, 1000 trials of running algorithms 2 and 4 were performed as described above for the error-free case, and the mean of the relative error in 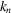 and the standard deviation were calculated across all 1000 trials [Figs. 1 ▸(*a*), 1 ▸(*b*)]. Additionally, the crystallographic *R* factor was calculated [Fig. 1 ▸(*c*)]. Both algorithms perform similarly up to the coordinate error of 0.4 Å, leading to the relative error under 20%; this coordinate error is within various estimates reported in the literature [see, for example, pp. 658–662 in Rupp (2009 ▸), and references therein]. After that limit, algorithm 2 performs systematically better. For large coordinate errors, using second derivatives of (6) explicitly calculated and supplied to L-BFGS

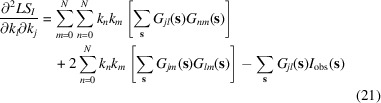

improved the performance of algorithm 2 further. Overall, algorithm 2 with second derivatives seems to perform best across all trials in terms of yielding the lowest relative error [Fig. 1 ▸(*a*)] and more consistently [Fig. 1 ▸(*b*)] compared with other algorithms. However, given that errors of magnitude 0.5 Å or larger are rather rare and unrealistic, and algorithm 2 is much slower than algorithm 4, the latter may be the default option of choice for practical applications.

### 3.4. Robustness with respect to the number *N* of components

In the tests above, the rather small number of components contributing to the total model structure factor (3–4) were defined by the atomic model of choice and remained the same in all calculations. However, the number and size (especially relative to the macromolecule and to each other) of these components can potentially affect the performance of the algorithms. To explore this, the following numeric experiment was set up. The lysozyme model (PDB code 1jkb, Muraki *et al.*, 1997 ▸) was obtained from the PDB (Burley *et al.*, 2021 ▸) and placed in the middle of a virtual *P*1 unit-cell box. The atomic model occupied 25% of the unit cell, which corresponds to a somewhat above average solvent content. Individual regions that contribute to the total model structure factor were mimicked by spheres placed in the solvent region of the unit cell such that they occupied the entire solvent region and did not overlap with the protein and themselves. The size (radius 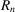 ) and occupancy 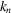 of each sphere were chosen randomly between 3 and 10 Å and 0.1 and 100, correspondingly. This typically generated between 30 and 50 spheres. Using spheres as individual mask components allowed a fast calculation of their structure factors analytically using the same *B* factor equal to 50 Å^2^ as in the previous tests:

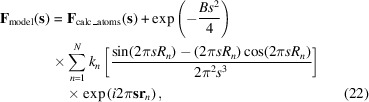

where 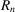 is the sphere radius and 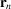 is its center (Appendix *A* [App appa]). The rest of the test was performed exactly as in the previous example and yielded essentially similar results (not shown).

The Python code of the numeric test described above is part of the *CCTBX* distribution and is used as a regression test for algorithms 2 and 4; it is located in the mmtbx.bulk_solvent module of *CCTBX*.

### 3.5. Errors in experimental data

**Figure 2:**
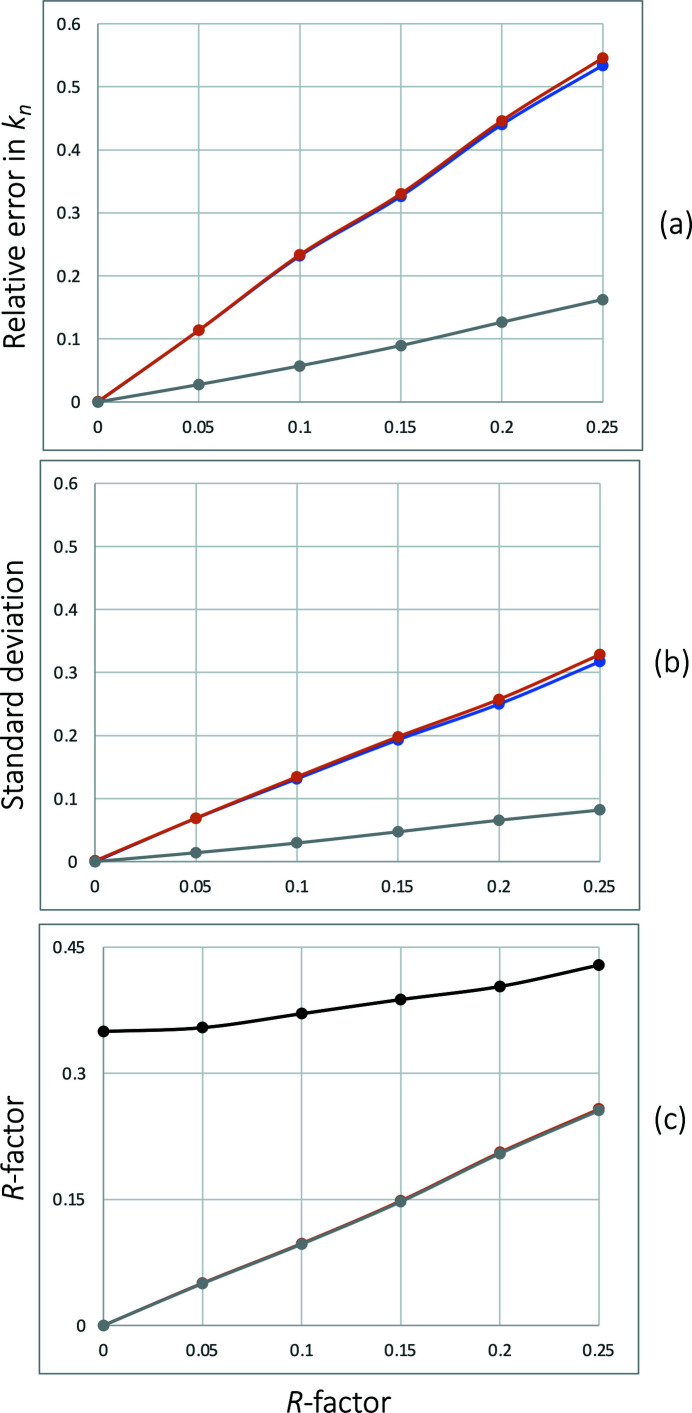
Relative mean error in 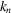 (*a*), its standard deviation (*b*) and (*c*) *R* factor between error-free simulated 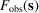 and 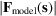 (20) computed using 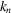 values recovered by algorithm 4 (gray), algorithm 2 without second derivatives (orange) and algorithm 2 using second derivatives (blue), plotted as a function of a random Gaussian error introduced to observed 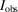 which is expressed through the respective *R* factor. The black line in (*c*) shows the initial *R* factor calculated assuming all 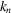 values are zero.

So far, all tests described above have been done using the model-simulated error-free experimental data. While modeling the experimental data which include many various sources of errors, *e.g.* those discussed by Borek *et al.* (2003 ▸) and Pozharski (2012 ▸), is a challenging task, here we focused on the simplest and most straightforward case of independent random errors distributed using the Gaussian law. These errors were introduced into model-calculated values of 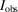 such that the resulting values of 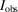 with errors match the exact error-free values up to specified *R* factors of 0 (no errors), 5, 10, 15, 20 and 25%. This mimics the typical *R*-factor values in macromolecular crystallography performed at a broad range of resolutions of the experimental data: from ultra-high to mid-low (*e.g.* Urzhumtsev *et al.*, 2009 ▸). Similarly to Section 3.3, 1000 runs were done for each of six error doses introduced to 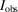 . In terms of robustness and consistency of recovering 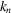 , algorithm 4 performed notably better than either of the two versions of algorithm 2 [Figs. 2 ▸(*a*), 2 ▸(*b*)]. This is likely because algorithm 4 uses model phases and in this test model phases were kept error free.

### 3.6. Test with real (not simulated) experimental data

**Figure 3:**
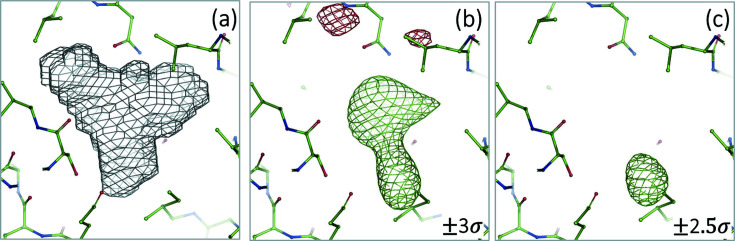
Bulk solvent mask (*a*) outlining a pocket inside the protein (PDB: 4gu0, near residue 131 in chain H) and the weighted difference map (Read, 1986 ▸) calculated assuming this pocket is empty (*b*) or filled with a solvent that was modeled using algorithm 4 (*c*). Map contouring levels are indicated on the figure.

For this test we have selected a model and experimental data from the PDB (PDB code: 4gu0, Chen *et al.*, 2013 ▸) and focused on an isolated region inside the protein near residue 131 in chain H [Fig. 3 ▸(*a*)]. The residual density map still shows a rather strong peak in this region [Fig. 3 ▸(*b*)] after solvent and all scales have been accounted for using the standard approach as implemented in *CCTBX* (Afonine *et al.*, 2013 ▸), which suggests that the region is occupied by either a disordered ligand or by a solvent other than the bulk solvent everywhere else. This region is considered as an independent component in (4) and its scale factor 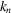 was obtained using both algorithms 2 and 4. The inclusion of this region in the total model structure factor (4) with refined 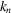 (both algorithms yielded virtually the same value) flattened out the residual density map [Fig. 3 ▸(*c*)].

## 4. Discussion

The multi-component approach to modeling the crystal content provides an opportunity for a more complete and accurate description. The model described here allows for explicit inclusion of semi-ordered solvent, disordered ligands and parts of the macromolecule as well as the features in the bulk solvent that deviate from the flat solvent model. In this approach each feature being modeled, which is not a part of the atomic model nor bulk solvent, is treated individually and its contribution to the total model structure factor is added as a correction term with a refinable resolution-dependent scale factor. Calculating these scale factors in a numerically efficient and stable manner is an algorithmic challenge to which we provide a solution. Algorithm 1 is the most straightforward in terms of implementation but at the same time it is the most runtime expensive and offers no guarantee of convergence to the correct result. Algorithm 3 does not require iterations and leads to the solution analytically; however, it is sensitive to rounding errors and is very computer memory expensive. While we found that both algorithm 2 (using second derivatives) and algorithm 4 perform almost identically in terms of recovering parameters in our tests with reasonable-size errors, algorithm 2 requires substantially more calculations and thus it is more runtime expensive. Therefore, algorithm 4 is suggested as the default choice. All the algorithms described here are implemented in *CCTBX* (mmtbx.bulk_solvent module) and are available in the *Phenix* suite starting from version 1.20rc4-4425. Putting these algorithms in production to automatically model non-uniform features of the bulk solvent and disordered parts of the atomic model, both macromolecule and ligands, is an ongoing effort within the *Phenix* team and collaborators.

## Appendix A

Let us define a binary mask 1/0 of a sphere at the origin and with radius *R*. Its Fourier transform (scattering function, or structure factors if we consider  as points of the reciprocal-space grid)

is spherically symmetric. Being expressed in spherical coordinates with  ,  and  the angle between the vectors  and  , its radial component

becomes equal to the 3D interference function times the volume of the integration sphere:
